# Drug Repositioning for Fabry Disease: Acetylsalicylic Acid Potentiates the Stabilization of Lysosomal Alpha-Galactosidase by Pharmacological Chaperones

**DOI:** 10.3390/ijms23095105

**Published:** 2022-05-04

**Authors:** Maria Monticelli, Ludovica Liguori, Mariateresa Allocca, Andrea Bosso, Giuseppina Andreotti, Jan Lukas, Maria Chiara Monti, Elva Morretta, Maria Vittoria Cubellis, Bruno Hay Mele

**Affiliations:** 1 Department Biology, University of Napoli « Federico II », Complesso Universitario Monte Sant’Angelo, Via Cinthia, 80126 Napoli, Italy; maria.monticelli@unina.it (M.M.); andrea.bosso@unina.it (A.B.); bruno.haymele@unina.it (B.H.M.); 2 Department Environmental, Biological and Pharmaceutical Sciences and Technologies (DiSTABiF), University of Campania “Luigi Vanvitelli”, Via Vivaldi 43, 81100 Caserta, Italy; lud.liguori@gmail.com (L.L.); mariateresa.allocca@unicampania.it (M.A.); 3 Institute of Biomolecular Chemistry ICB, CNR, Via Campi Flegrei 34, 80078 Pozzuoli, Italy; gandreotti@icb.cnr.it; 4 Institute of Biochemistry and Cellular Biology, National Research Council, Via Pietro Castellino 111, 80131 Napoli, Italy; 5 Translational Neurodegeneration Section “Albrecht-Kossel”, Department of Neurology, University Medical Center Rostock, 18147 Rostock, Germany; jan.lukas@med.uni-rostock.de; 6 Center for Transdisciplinary Neurosciences Rostock (CTNR), University Medical Center Rostock, 18147 Rostock, Germany; 7 Department of Pharmacy, University of Salerno, Via Giovanni Paolo II 132, 84084 Fisciano, Italy; mcmonti@unisa.it (M.C.M.); emorretta@unisa.it (E.M.)

**Keywords:** drug repositioning, pharmacological chaperones, acetylsalicylic acid, AGAL, Fabry disease, lysosomal storage diseases

## Abstract

Fabry disease is caused by a deficiency of lysosomal alpha galactosidase and has a very large genotypic and phenotypic spectrum. Some patients who carry hypomorphic mutations can benefit from oral therapy with a pharmacological chaperone. The drug requires a very precise regimen because it is a reversible inhibitor of alpha-galactosidase. We looked for molecules that can potentiate this pharmacological chaperone, among drugs that have already been approved for other diseases. We tested candidate molecules in fibroblasts derived from a patient carrying a large deletion in the gene *GLA,* which were stably transfected with a plasmid expressing hypomorphic mutants. In our cell model, three drugs were able to potentiate the action of the pharmacological chaperone. We focused our attention on one of them, acetylsalicylic acid. We expect that acetylsalicylic acid can be used in synergy with the Fabry disease pharmacological chaperone and prolong its stabilizing effect on alpha-galactosidase.

## 1. Introduction

Fabry disease (FD) is caused by a deficiency of the enzyme lysosomal alpha-galactosidase (AGAL) that removes a terminal galactose residue from globotriaosylceramides such as Gb3. It is encoded by the gene *GLA* on the X chromosome [1,2,3,4,5].

FD exhibits a large spectrum of phenotypes, ranging from severe early-onset forms to atypical or mild late-onset forms, and is associated with more than 1000 genotypes, none of which are prevalent [6,7,8,9].

Contrary to what was observed in other X-linked diseases, FD affects heterozygous females as well as hemizygous males [10,11,12]. Patients who do not produce AGAL require enzyme replacement therapy (ERT). ERT consists of repeated intravenous infusions of a recombinant AGAL [13]. Patients who carry a missense mutation in *GLA* may benefit from oral therapy with a pharmacological chaperone (PC) [14], but, unfortunately, not every patient is eligible. Since hundreds of different genotypes are known, a large effort was put in place to predict and experimentally validate which missense variants are treatable with PCs [15,16]. In only a few cases were these experiments carried out ex vivo, in fibroblasts or leucocytes, due to the difficulty of obtaining cells from patients [17,18,19,20]. In most cases, experiments were carried out with vectors containing the *GLA* gene variants for heterologous expression in COS1, COS7, or HEK293 cells [9,21,22,23,24,25,26,27,28]. PCs can bind and stabilize mildly-destabilizing variants (most often caused by missense mutations in the flexible, exposed regions of the protein) [29] but cannot rescue severely-destabilizing mutations and those affecting the active site or disulfide bridges [30]. 1-Deoxygalactonojirimycin (DGJ), also known as migalastat, is an imino sugar analog of galactose that stabilizes wild-type AGAL as well as amenable (i.e., responsive) variants [31]. It binds the active site and acts specifically as a competitive inhibitor. Although a few attempts have been carried out to identify a molecule that stabilizes AGAL without inhibiting it in the lysosomes [32,33], DGJ is the only approved PC for FD so far and is marketed under the name Galafold (Amicus Therapeutics, Philadelphia, PA, USA) [34]. PCs and ERT are not a cure for FD but chronic treatments. DGJ requires a precise dosage and an intermittent regimen where the stabilizing and inhibitory effects are carefully balanced [17,35,36,37]. Combining DGJ with other drugs that raise activity of AGAL variants by different mechanisms would ameliorate the therapy [38].

AGAL is synthesized as a high molecular weight precursor, imported into the endoplasmic reticulum, transferred to the lysosome, and selectively released extracellularly [39]. The enzyme undergoes maturation, which includes proteolysis, glycosylation, and phosphorylation on mannose residues [40,41]. Unstable variants are cleared by the quality control system and their concentration, and consequently the total activity in the cell, is lower than normal. Several pathways contribute to proteostasis and could represent the source of novel targets for the treatment of FD, as was proposed for other lysosomal storage disorders [42,43]. A paper by Seemann et al. described the screening of proteostasis regulators as potentiators of DGJ. The authors found that clasto-lactacystin β-lactone, MG132, and bortezomib enhance the effect of DGJ in two lines of fibroblasts derived from Fabry patients [44]. In the same cellular model, they could not confirm the positive effect of ambroxol (ABX), which had been previously observed in transiently transfected cells [45]. An effector of heat-shock proteins, 4-phenyl-butyrate (4-PBA), was tested in fibroblasts derived from FD patients in monotherapy. The drug raised the amount of intracellular AGAL precursor but did not enhance the enzymatic activity [46]. The mode of action of proteostasis and heat-shock regulators is not as straightforward as one would predict. For example, bortezomib affects proteostasis as well as *GLA* expression [45], and 4-PBA acts as a chemical chaperone binding hydrophobic patches of misfolded proteins [47] and controls ER stress. Disentangling their precise role can be difficult [48].

We propose a practical approach based on repositioning, in which the effects of drugs on total AGAL activity are tested in synergy with DGJ in a suitable cellular model. Transient transfection of a plasmid expressing AGAL mutants in COS or HEK cells is very useful for PCs because any missense mutation can be tested. However, this may cause unforeseen artificial results due to overexpression, e.g., an unphysiological overload of the ER protein folding apparatus not observed in cells even in pathological conditions. We propose a cellular model where the advantages of FD-derived cells and the versatility of transfection are combined. We produced stably transfected fibroblasts derived from an FD patient carrying a large deletion to test FDA-approved drugs in synergy with DGJ. We started with 4-PBA and ABX, then moved on to other DGJ potentiators, looking for safe and cheap drugs that can be used for life-long chronic treatment.

We found that acetylsalicylic acid(ASA) can raise the total amount and activity of AGAL in synergy with DGJ on amenable mutations.

## 2. Results

### 2.1. Establishment of Versatile Cell Models That Do Not Overexpress AGAL

DGJ is routinely tested on different missense variants in transiently transfected HEK293 cells [9,15]. While wt-*GLA* is present in the genome of these cells, the effect of the drug can be evaluated regardless, as the missense mutants are overexpressed.

In a recent review [49], some drawbacks of this method were pointed out, such as the major concerns involving the endogenous AGAL activity of wild-type HEK293 cells and the overexpression of artificial constructs. The first issue can give false positive results when variants exhibit very low residual activities. Lenders et al. developed a method based on transient transfection of CRISPR-Cas9-mediated *GLA*-knockout HEK293T cells [50]. They brilliantly solved the problem of endogenous AGAL activity, and they demonstrated that some variants previously classified as amenable were actually non-amenable.

**Figure 1:**
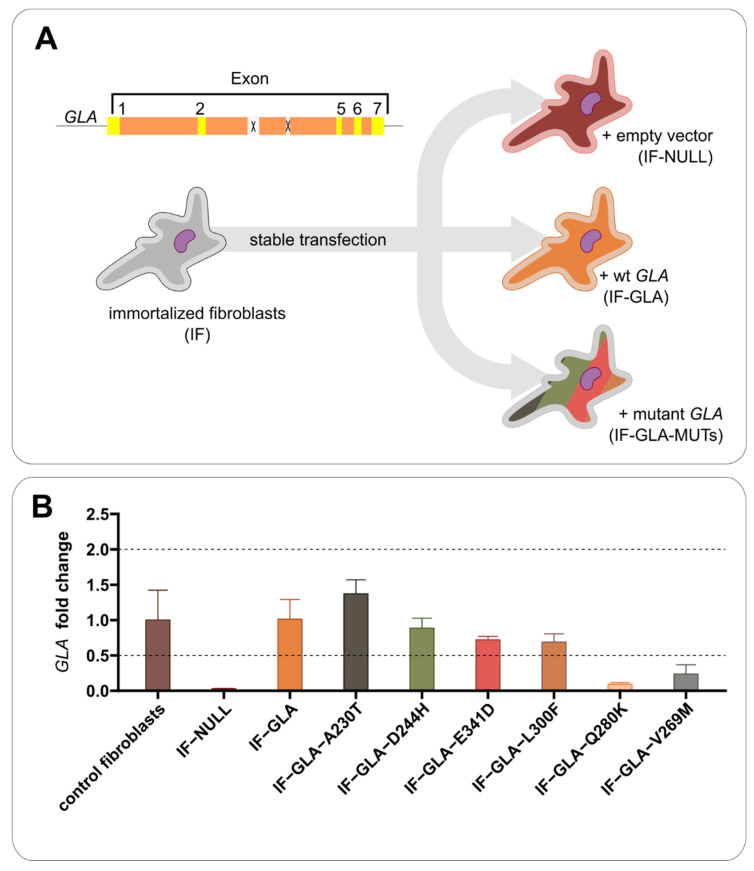
Cell line establishment. Fabry patient’s fibroblasts carrying a deletion of exons 3 and 4 in the *GLA* gene were obtained from a Telethon biobank. They were immortalized (IF), then stably transfected with an empty vector (IF-NULL), the wt-*GLA* (IF-GLA), or different *GLA* pathogenic mutants (IF-GLA-MUTs) (**A**). *GLA* expression (RNA) was measured by RT-qPCR (*n* = 3); the selected cell lines did not over-express *GLA* compared to healthy fibroblasts (**B**).

We felt that testing proteostasis regulators under conditions of super-expression was not appropriate. Nonetheless, the versatility of transfected cells is needed to test any possible missense variant. It was for this reason that we stably transfected a line of fibroblasts derived from a male FD patient carrying a large deletion of exons 3 and 4 in *GLA*. The cells were immortalized (IF cells) as described in the Methods section. Starting from a single clone, IF cells were stably transfected with a plasmid encoding wt-*GLA*, obtaining IF-GLA cells, or encoding hypomorphic *GLA* mutants, obtaining IF-GLA-MUTs, or with an empty vector, obtaining IF-NULL cells (Figure 1A). We chose missense mutations that do not affect the active site, do not prevent folding, and that were previously tested by our group with cells transiently transfected [25].

Transcriptional levels of *GLA* in the cell lines were comparable to or lower than those in healthy fibroblasts (Figure 1B).

Our models are artificial since the promoter is not the same of *GLA* and an intron-less gene is encoded in our constructs. Moreover, CRISPR-Cas9-mediated *GLA*-knockout HEK293T cells might replace the immortalized IF-NULL fibroblasts. However, a comparison with the literature confirmed the absence of overexpression possibly due to the lower copy number of plasmids attained with stable compared to transient transfection. As shown in Supplementary File S1 (S1), enzymatic activity in our cell lines was much lower than in transiently transfected cells [15]. Moreover, the wt-activity of our IF-GLA cells was comparable to that of healthy fibroblasts described by Seeman et al., 2020 and Benjamin et al., 2009 [17,44].

### 2.2. Intracellular Stabilization of AGAL by DGJ Is Enhanced by Ambroxol and 4-Phenylbutyrate

**Figure 2:**
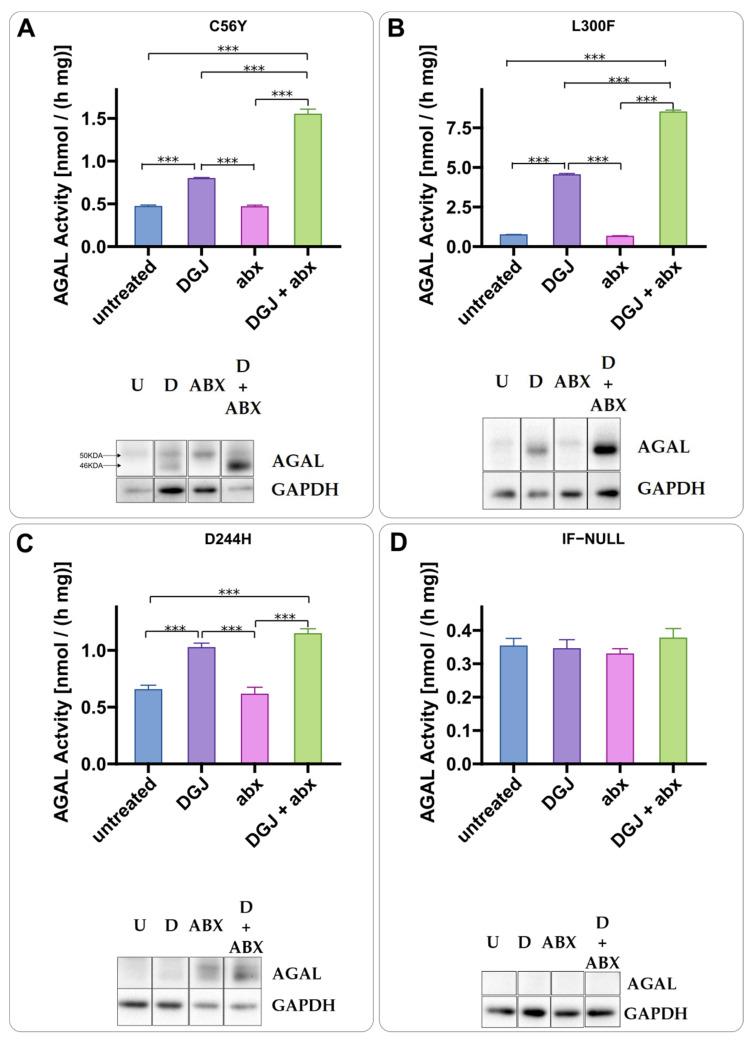
Ambroxol is a PC-enhancer. IF-GLA-C56Y (**A**), IF-GLA-L300F (**B**), and IF-GLA-D244H (**C**) were treated for 72 h with the following drugs: i. untreated; ii. 10 μM DGJ; iii. 40 μM ambroxol (ABX); iv. 10 μM DGJ + 40 μM ABX. AGAL specific activity measured on protein extracts is shown. Tukey’s HSD was used to evaluate significative differences among treatments (***: *p* < 0.001 *n* = 3). The effects of combined treatment (DGJ + ABX) are significantly larger than those of DGJ monotherapy in all the mutants except for D244H. Immunoblots confirmed the results (U = untreated; D = DGJ 10 μM; ABX = ambroxol 40 μM; D + ABX = DGJ 10 μM + ambroxol 40 μM). Arrows on the immunoblot in panel A highlight the higher molecular weight band (precursor) and the lower molecular weight band (active form) of AGAL. IF-NULL was used as a negative control (**D**). Each panel includes specific activity and an immunoblot for a cell line.

**Figure 3:**
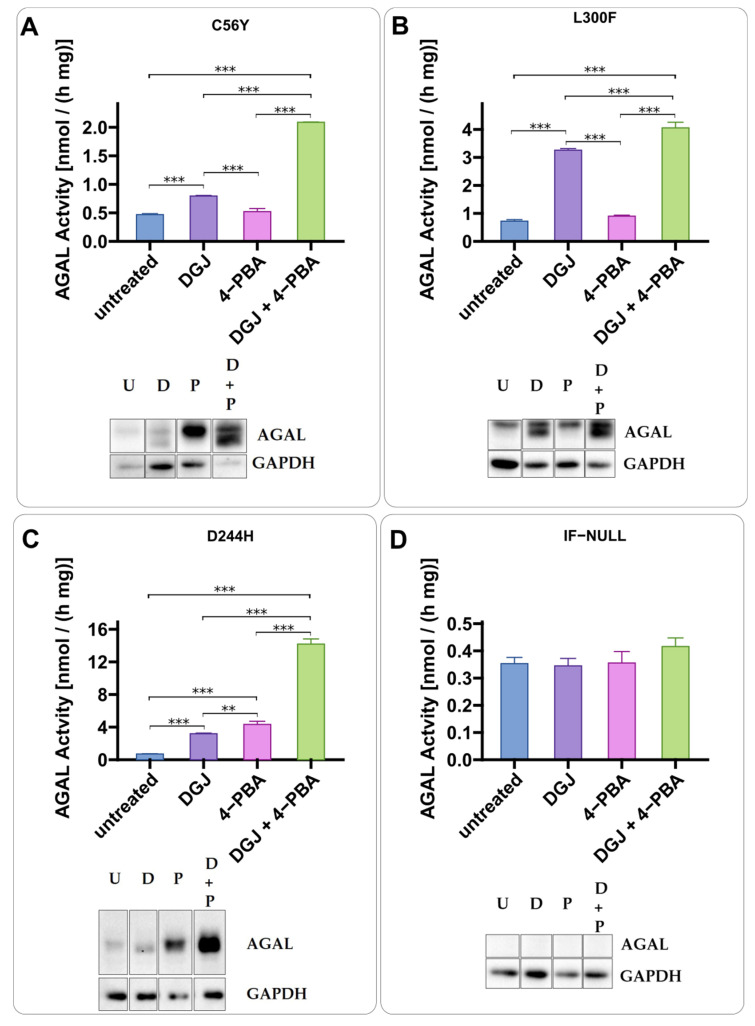
4-phenylbutyrate is a PC-enhancer. IF-GLA-C56Y (**A**), IF-GLA-L300F (**B**), and IF-GLA-D244H (**C**) were treated for 72 h with the following drugs: i. untreated; ii. 10 μM DGJ; iii. 4 mM 4-phenylbutyrate (4-PBA); iv. 10 μM DGJ + 4 mM 4-PBA. AGAL specific activity measured on protein extracts is shown. Tukey’s HSD was used to evaluate significative differences among treatments (***: *p* < 0.001; **: *p* < 0.01; *n* = 3). The effects of combined treatment (DGJ + 4-PBA) are significantly larger than those of DGJ monotherapy. Immunoblots confirmed the results (U = untreated; D = DGJ 10 μM; P = 4-PBA 4 mM; D + P = DGJ 10 μM + 4-PBA 4 mM). IF-NULL was used as a negative control (**D**). Each panel includes specific activity and an immunoblot for a cell line.

Ambroxol (Figure 2A,B) and phenylbutyrate (4-PBA) (Figure 3A,B) were tested in IF-GLA-MUTs. Both drugs enhanced the stabilizing effect of DGJ. A different mechanism of action with respect to DGJ was revealed by an immunoblot. The presence of the PC-enhancer alone increases AGAL precursor levels (higher molecular weight band) while the chaperone stabilizes the active form (lower molecular weight band). In general, the combined treatment results in a strong increase in the active AGAL. Figure 2 highlights the different molecular weights of the AGAL precursor and active form as an example.

### 2.3. Intracellular Stabilization of AGAL by DGJ Is Enhanced by Acetylsalicilic Acid

**Figure 4:**
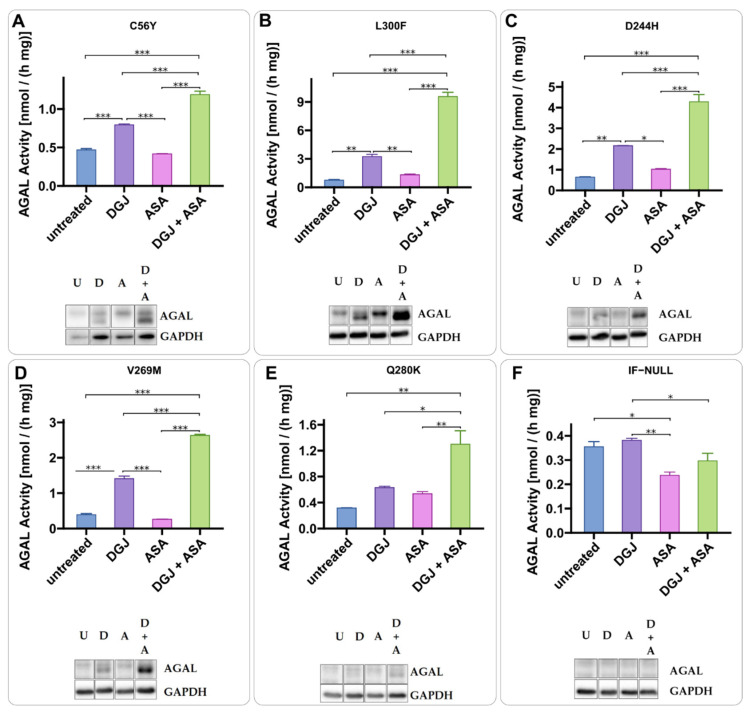
Acetylsalicylic acid is a PC-enhancer. IF-GLA-MUTs, specifically C56Y (**A**), L300F (**B**), D244H (**C**), V269M (**D**), and Q280K (**E**), were treated for 72 h with the following drugs: i. untreated; ii. 10 μM DGJ; iii. 4 mM acetylsalicylic acid (ASA); iv. 10 μM DGJ + 4 mM ASA. IF-NULL was used as a control (**F**). AGAL specific activity measured on protein extracts is shown. Tukey’s HSD was used to evaluate significative differences among treatments (***: *p* < 0.001; **: *p* < 0.01; *: *p* < 0.05; *n* = 2). The effects of combined treatment (DGJ + ASA) are significantly higher than those of DGJ monotherapy. Immunoblots confirmed the results (U = untreated; D = DGJ 10 μM; A = ASA 4 mM; D + A = DGJ 10 μM + ASA 4 mM). Each panel includes specific activity and an immunoblot for a cell line.

**Figure 5:**
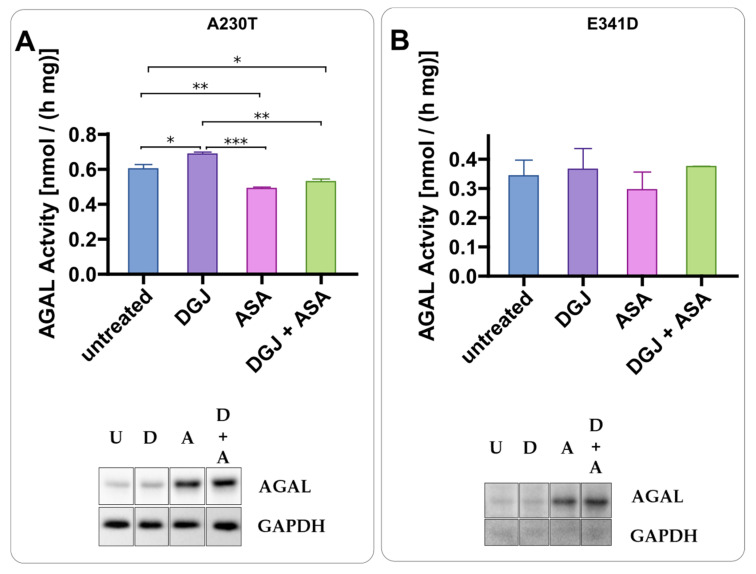
Effect of acetylsalicylic acid on non-amenable variants. IF-GLA-MUTs, specifically IF-GLA-A230T (**A**) and IF-GLA-E341D (**B**), were treated for 72 h with the following drugs: i. untreated; ii. 10 μM DGJ; iii. 4 mM acetylsalicylic acid (ASA); iv. 10 μM DGJ + 4 mM ASA. AGAL specific activity measured on protein extracts is shown. Tukey’s HSD was used to evaluate significative differences among treatments (***: *p* < 0.001; **: *p* < 0.01; *: *p* < 0.05; *n* = 2). As expected, the combined treatment did not increase the enzymatic activity of non-amenable variants. Immunoblots showed the increase of the AGAL precursor as a result of the acetylsalicylic acid treatment (U = untreated; D = DGJ 10 μM; A = ASA 4 mM; D + A = DGJ 10 μM + ASA 4 mM). Each panel includes specific activity and an immunoblot for a cell line.

To find useful PC enhancers among drugs that have been used for chronic treatment of patients for a long time, we tested acetylsalicylic acid (ASA). AS enhances the DGJ stabilizing effect. The presence of ASA increases AGAL precursor levels (higher molecular weight band) while the chaperone stabilizes the active form (lower molecular weight) (Figure 4). The combined treatment results in a strong increase in the active AGAL as already observed with ABX and 4-PBA. We also tested two variants, namely A230T and E341D, that do not affect the active site or prevent folding in principle but have been judged non-amenable on the basis of standard tests carried out on transiently transfected cells [25]. As shown in Figure 5, ASA increased the precursor level of both mutants, but DGJ could not promote the maturation. We did not identify any increase in activity following the combined treatment on these non-amenable variants (Figure 5).

**Figure 6:**
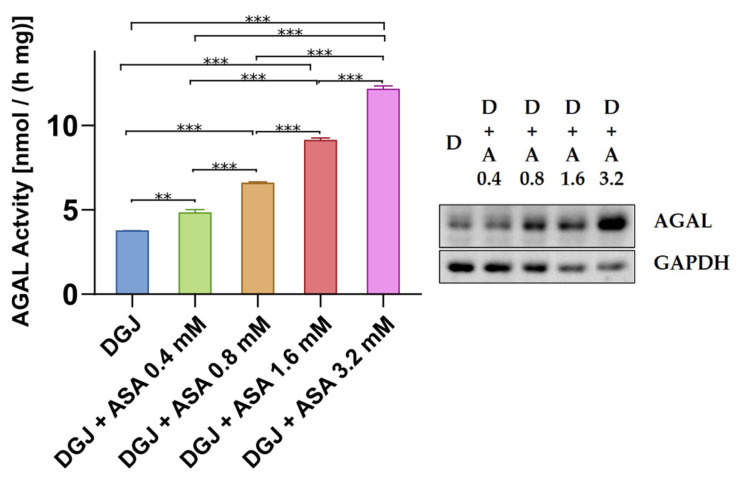
Acetylsalicylic acid effect is dose-dependent. IF-GLA-L300F was treated with different doses of acetylsalicylic acid (range 0–3.2 mM) in the presence of 10 μM DGJ. AGAL specific activity measured on protein extracts is shown. Tukey’s HSD was used to evaluate significant differences among treatments (***: *p* < 0.001; **: *p* < 0.01; *n* = 2). AGAL activity increase upon combined treatment is dose-dependent. Immunoblots confirmed the results (D = DGJ 10 μM; D + A = DGJ 10 μM + ASA).

The effect of ASA is dose-dependent (Figure 6).

**Figure 7:**
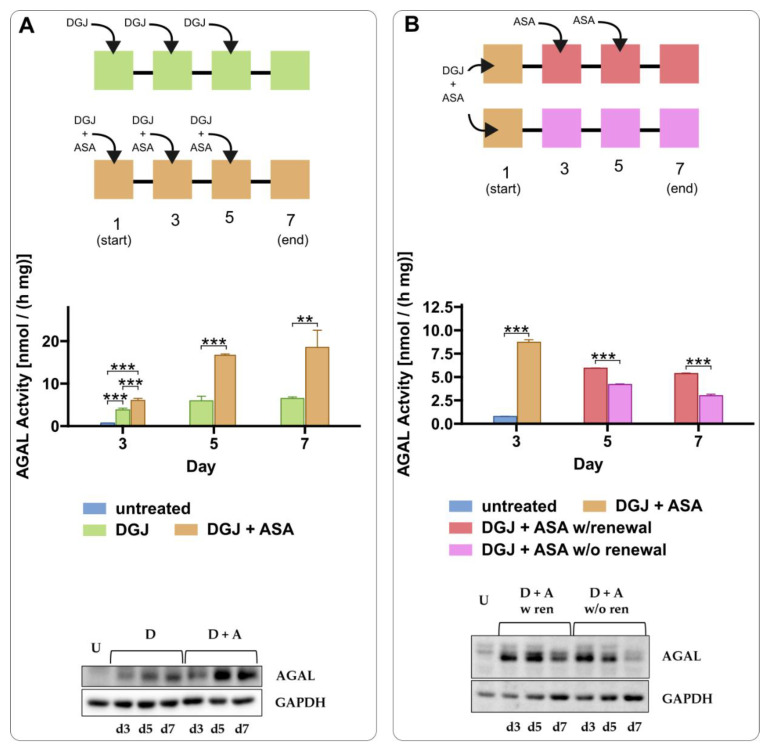
Acetylsalicylic acid prolongs the effects of DGJ. IF-GLA-L300F cells were treated with different combinations of 10 μM DGJ and 4 mM acetylsalicylic acid (ASA), and the AGAL content was evaluated for seven days. (**A**) compares DGJ monotherapy versus the combined therapy with ASA; drugs were renewed every other day. In (**B**), the effect of ASA renewal every other day was compared to the single-dose treatment. AGAL specific activity measured on protein extracts is reported. Tukey’s HSD was used to evaluate significant differences among treatments only between treatments within the same day (***: *p* < 0.001; **: *p* < 0.01; *n* = 3). No comparison between treatments belonging to different days was made. Immunoblot analysis confirmed the results (U = untreated; D + A w ren = DGJ 10 μM + ASA 4 mM with ASA renewal; D + A w/o ren = DGJ 10 μM + ASA 4 mM without ASA renewal). Each panel includes the experimental design, specific activity, and an immunoblot for each experiment.

The effect of ASA is observed when DGJ is administered every other day (Figure 7A) repeatedly. Interestingly, ASA prolongs the effects of DGJ, suggesting that a less frequent administration of the drug could be considered in patients. We tested a regimen in which DGJ + ASA were administered once a week with or without a booster of ASA every other day. The experimental design is sketched in Figure 7A,B.

**Figure 8:**
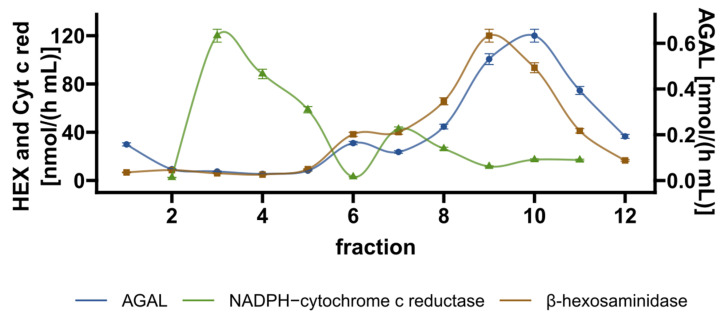
AGAL is localized in the lysosomes upon combined treatment. IF-GLA-L300F cells were treated with 10 μM and 4 mM ASA for 72 h. Subcellular particles were separated on a density gradient. The fractions were then analyzed by measuring the following activities: AGAL, β-hexosaminidase (HEX, a lysosomal marker), and NADPH-cytochrome c reductase (Cyt c red, an ER marker). Fractions enriched in AGAL were also enriched in HEX but not in Cyt c red, which showed its peak in distinct fractions.

IF-GLA-L300F was treated for 72 h with DGJ + ASA. Cell content was fractionated on a density gradient. Fractions were analyzed by enzyme activities to identify the subcellular particles. AGAL co-localizes with the lysosomal marker β-hexosaminidase and it does not co-localize with the ER marker NADPH-cytochrome c reductase. The experiment was carried out twice and Figure 8 shows one representative result.

**Figure 9:**
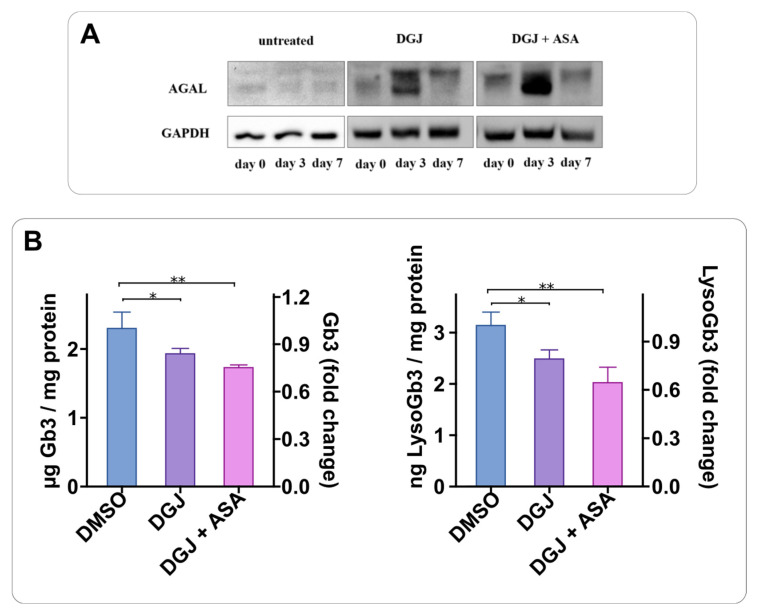
Combined long-term treatment of DGJ and ASA reduces Gb3 and LysoGb3 in cells. In (**A**), a pilot analysis via immunoblot shows that 7 days are required to exhaust the effect of drugs. In (**B**), IF-GLA-L300F cells were treated with 10 μM DGJ with or without 4 mM ASA every 7 days, up to 50 days; drug administration timing was based on the pilot experiment; a control was performed in the absence of drugs (DMSO). (**B**) displays the content of Gb3 and LysoGb3 on day 50. The cells were collected, and the amount of Gb3 and LysoGb3 was measured with LC-MS/MS. Tukey’s HSD was used to evaluate significant differences among treatments (**: *p* < 0.01; *: *p* < 0.05; *n* = 3).

We tested the effect of the combined therapy on Gb3 and Lyso-Gb3 accumulation. To validate the benefit of the combined treatment, we explored its effect on substrate reduction over a long-term treatment. To this end, we performed a pilot screen and established the timing to be used. Figure 9A shows that seven days are required to exhaust the effect of the drugs. Thus, we administered the drugs DGJ or DGJ + ASA once a week for fifty days, and then evaluated the Gb3 and LysoGb3 content in the cells. As for the Gb3, the most abundant forms were considered, namely C16:0, C24:0, and C24:1. A representative chromatogram of the Gb3 isoforms contained in these samples is shown in Supplementary File S2 (S2). As shown in Figure 9B, both DGJ and DGJ+ ASA significantly reduce the amounts of Gb3 and Lyso-Gb3.

### 2.4. Mode of Action of Acetylsalicylic Acid

**Figure 10:**
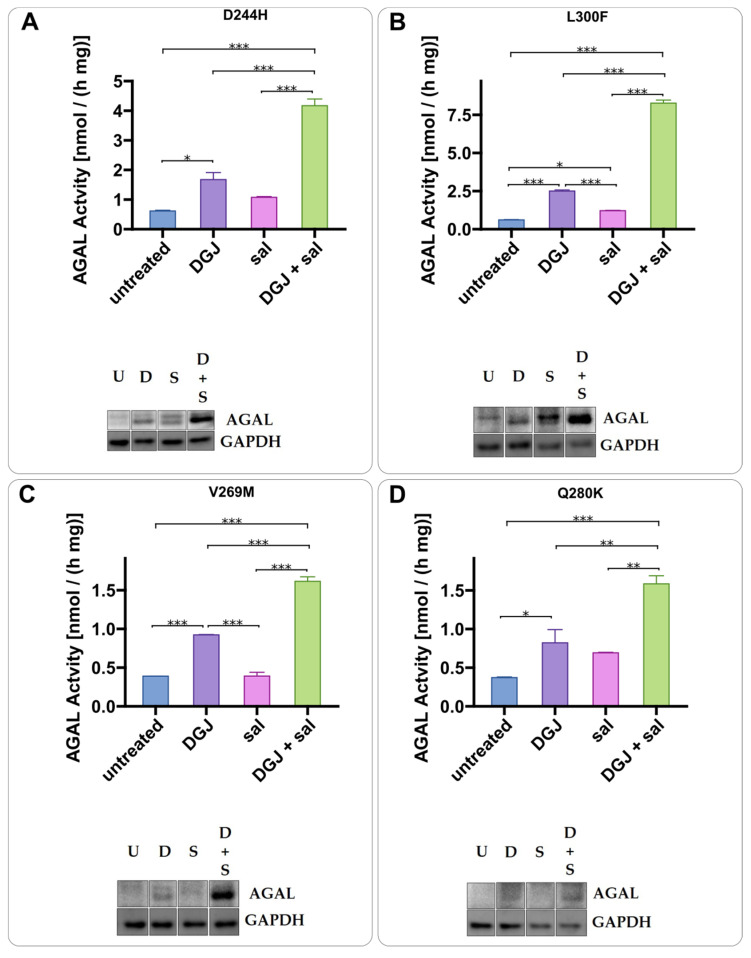
Salicylic acid acts as acetylsalicylic acid. IF-GLA-MUTs, specifically D244H (**A**), L300F (**B**), V269M (**C**), Q280K (**D**, were treated for 72 h with the following drugs: i. untreated; ii. 10 μM DGJ; iii. 4 mM salicylic acid (sal); iv. 10 μM DGJ + 4 mM sal. AGAL specific activity measured on protein extracts is shown. Tukey’s HSD was used to evaluate significative differences among treatments (***: *p* < 0.001; **: *p* < 0.01; *: *p* < 0.05; *n* = 2). The effects of combined treatment (DGJ + sal) are significantly higher than those of DGJ monotherapy. Immunoblots confirmed the results (U = untreated; D = DGJ 10 μM; S = sal 4 mM; D + S = DGJ 10 μM + sal 4 mM). Each panel includes specific activity and an immunoblot for a cell line.

ASA can acetylate a large range of cellular proteins [51] and in so doing it prevents protein aggregation in certain cases [52]. This does not appear to be the case since AGAL stabilization in synergy with DGJ can be obtained using salicylate (Figure 10).

**Figure 11:**
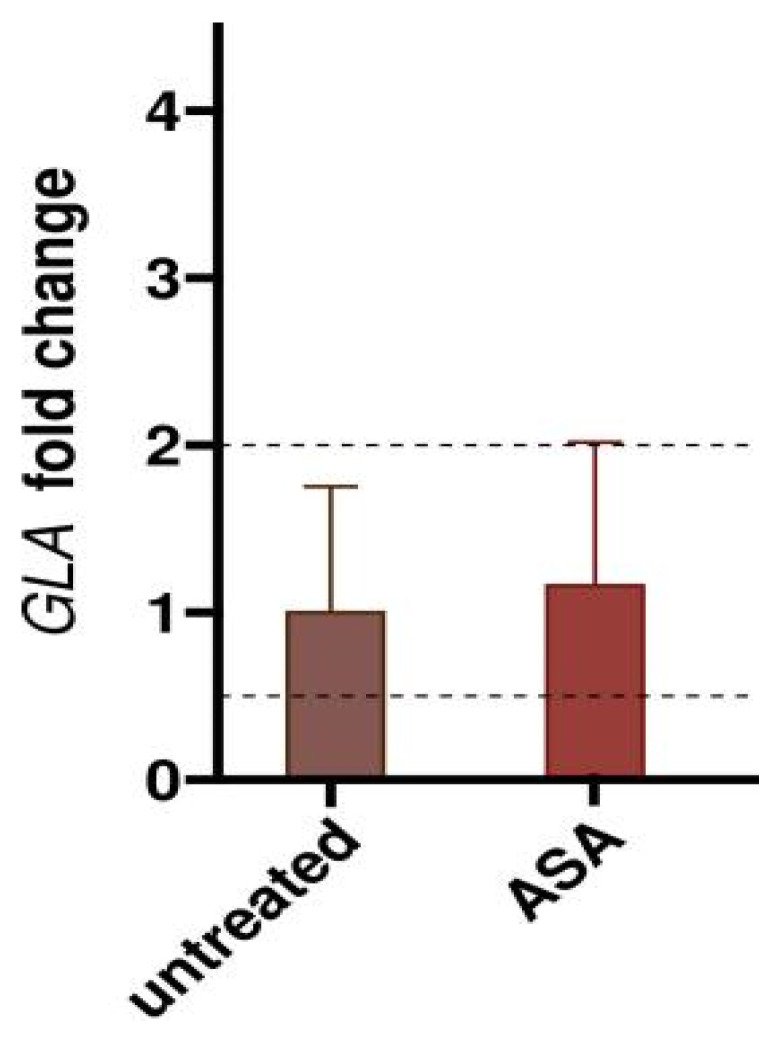
The effect of acetylsalicylic acid is not transcriptional. IF-GLA-L300F cells were treated with 4 mM ASA for 72 h. *GLA* expression was measured by RT-qPCR (*n* = 3).

The positive effect of ASA in our cells is not caused by transcriptional changes, as shown in Figure 11.

ASA does not inhibit AGAL (data not shown). This finding does not exclude the possibility that ASA binds allosterically without interfering with the enzymatic activity.

To assess the indirect effects of ASA on AGAL stabilisation, we carried out a proteomics analysis of IF-GLA-L300F [53]. The analysis highlighted 292 proteins for which ASA treatment resulted in differential abundance (Supplementary File S3). Within this set, 148 were significantly (adj. *p*-value ≤ 0.1) less abundant in the ASA treatment vs. the control (FC ≤ 0.75), while 144 showed the opposite trend (FC ≥ 1.25).

AGAL was significantly more abundant in the ASA treatment (FC 2.41, adjusted *p*-value ≤ 6.16 × 10^−2^, following what was observed in Figure 4, panel B.

SNARE-associated protein Snapin, COG complex subunit 5 (COG5), and vacuolar-sorting protein SNF8 (SNF8), are the proteins most affected by ASA treatment.

Snapin was 12.4 times more abundant in ASA (adj. *p* ≤ 1.34 × 10^−10^).

COG5 and SNF8 were strongly downregulated in ASA-treated cells (FC 0.103 and 0.189, respectively, with adj. *p* ≤ 2.52 × 10^−16^).

Caveolin-1 (CAV1) was also upregulated (FC 2.06 with adj. *p* < 4.06 × 10^−4^).

Finally, two chaperones, heat shock 70 kDa protein 1-like (HSPA1L) and DnaJ homolog subfamily B member 4 (DNAJB4), were significantly upregulated in cells treated with ASA (FC 3.29 and 1.74, with adj. *p* ≤ 5.81 × 10^−3^ and 7.61 × 10^−2^, respectively).

ASA’s regulated expression of several genes was reported in the Expression Omnibus (GEO) repository. We analyzed the GSE58162 series because the data had been obtained under experimental conditions (2.0 mM ASA and 72 h of treatment) similar to those employed by us. Differential expression analysis of this dataset highlighted 1192 DEGs (*p* ≤ 0.05) (Supplementary File S4). 

The proteomic and transcriptomic results do not overlap. This discrepancy is expected, and attributable to the translational efficiency and different half-lives of mRNA and proteins [54,55,56,57,58]. Furthermore, the cell systems analyzed in the GSE58162 series was different from the cell system we treat here. Interestingly, the transcriptomic data indicate that the expression of several genes encoding proteasome-associated proteins and molecular chaperones is affected by ASA.

Eighteen interactions between ASA and human proteins are reported in the Biological General Repository for Interaction Datasets (BioGRID) [59]. Most of the reported proteins are involved in inflammation; among them, we found a protein involved in folding and quality control: heat shock 70 kDa protein 5, also known as BiP. This interaction has been analyzed by Deng et al. [60].

Two of these ASA BioGRID interactors (AKR1C1 and NFKB1) displayed a significant upregulation in ASA-treated cells (FC 1.82 and 2.09, adj. *p* ≤ 5.26 × 10^−4^ and 6.88 × 10^−4^, respectively) in the proteomic analysis.

## 3. Discussion

In this paper, we present a case of drug repositioning for the treatment of rare diseases. This approach has been proposed by many authors [61,62,63]. Indeed, iminosugars for the treatment of lysosomal storage disorders are themselves a successful example of drug repositioning since they had been planned as antiviral agents for the cure of HIV [64].

Finding potentiators of DGJ was our aim. We employed two drugs already tested for FD, namely ambroxol and 4-PBA, and proved that they work in co-administration with DGJ in cells stably transfected with *GLA* variants.

We moved to ASA, a molecule widely employed in the chronic management of different pathologies. In the case of FD patients, it is often associated with specific treatments, such as ERT for stroke prophylaxis [65,66]. Our results highlighted a novel role of ASA, that is, its ability to enhance the stabilizing effect of DGJ on amenable *GLA* mutants.

Our findings showed the increased stabilization of AGAL mutants upon combined treatment with DGJ and ASA. The mature active form is the one that accumulates in lysosomes. Interestingly, the presence of ASA prolonged the stabilization of AGAL over time, pointing towards a re-modulation of the therapy’s timing. Furthermore, a reduction in the accumulation of both Gb3 and LysoGb3 was observed after long-term combined treatment.

We are aware of the limitations of our study given that it was conducted in a cellular model in vitro. Adverse effects such as bleeding or gastric mucosal damage can occur in patients with a high dosage of ASA. In humans, 3 g orally per day in divided doses can be used with 1.1–2.2 mM target plasma salicylate levels [67,68].

Often a new use for an old drug is found serendipitously, and the mechanism by which a molecule designed for a specific target works on a different one is not clear.

To deepen our understanding of the effect of ASA on Fabry, we treated an AGAL-deficient cell line (IF-L300F) with ASA and compared its proteome with the untreated cell line. Notably, ASA-treated cells were significantly enriched in AGAL, as highlighted by immunoblot experiments.

Fabry disease is a lysosomal storage disorder, and proteins involved in membrane formation and trafficking are the most affected by treatment with ASA.

The SNARE-associated protein Snapin, encoded by the *SNAPIN* gene, is strongly upregulated in ASA-treated cells.

Snapin is a component necessary for the biogenesis of LRO (lysosome-related organelles). It is heavily involved in intracellular vesicle trafficking [69] and contributes to lysosome movement [70].

COG5 (conserved oligomeric Golgi complex subunit 5) is required to control Golgi structure and function as part of the Conserved oligomeric Golgi complex [71].

SNF8 (vacuolar-sorting protein SNF8) is required for sorting endosomal cargo proteins into late endosomes (multivesicular bodies).

We found that caveolin-1 was upregulated. This protein is one of the fundamental constituents of Golgi-derived transport vesicles and is known to be downregulated in Fabry mouse aortic endothelial cells [72].

The evaluation of differential protein expression in our cell line confirmed the effect of ASA through the differential expression of two known ASA interactors.

NFKB (NF-kappa-B) is a well-known transcription factor involved in the terminal phase of signal transduction associated with many biological processes.

AKR1C1 (aldo-keto reductase family 1 member C1) plays a crucial role in the progesterone metabolism and other steroid hormones [73].

Our experiment also suggests that ASA regulates the expression of molecular chaperones, with two upregulated genes.

The heat shock 70 kDa protein 1-like is implicated in the folding and transport of new polypeptides and is associated with the proteolysis of misfolded proteins, including targeting proteins for degradation [74]. DnaJ homolog subfamily B member 4 is a co-chaperone of the J protein family that has been observed to promote refolding [75].

The proteomics analysis of ASA-treated AGAL-deficient cells highlighted the capacity of ASA in regulating molecular chaperones. Our hypothesis is that ASA raises the amount of AGAL precursors, whose conversion in the active form is promoted by DGJ. This could explain the synergy between the two drugs.

Understanding the mechanism by which ASA works requires specific experiments. The results obtained by the proteomics analysis will allow focusing these experiments on certain proteins. Our data, which were obtained in a cell model that expresses an unstable variant of AGAL, support the idea that heat shock proteins and their interactors DNAJ facilitate the folding of proteins [76]. The role of the heat shock 70 kDa protein 1-like is particularly intriguing because it has been demonstrated that recombinant HSP70 improves the binding of AGAL (*GLA*), but also of α-galactosidase B (*GLB1*), neuraminidase (*NEU1*), arylsulfatase A (*ARSA*) and ß-hexosaminidase A (*HEXA*) to their co-factor, bis(monoacylglycero)phosphate [77]. The action of the molecular chaperone would not be specific for AGAL, but in general for lysosomal enzymes. In the case of acid beta-glucosidase (*GBA*), the induction of HSP70 enhances the folding, maturation, activity, and correct cellular localization of variants responsible for Gaucher disease [78].

Further studies will be needed to fully elucidate the mechanism of action of ASA.

## 4. Conclusions

DGJ was approved by the FDA for use on amenable mutations [79]. Several positive reports have accumulated since its approval [80,81,82]. Nonetheless some concerns have been raised regarding its effectiveness [14,83]. It should not be underestimated that DGJ inhibits AGAL at neutral and acidic pH, and it is unlikely that it stabilizes the enzyme in the ER and does not inhibit it in the lysosome [31]. For this reason, continuous administration of the drug as well as over-dosage is counterproductive. Several regimens were tested in a mouse model and in cell models. It was demonstrated that an intermittent administration of DGJ is more effective than daily administration in terms of substrate reduction [17,37]. Presently, an intermittent regimen is adopted in patients, and 150 mg of the drug is administered orally every other day. In this paper, we showed that it is possible to combine drugs to potentiate the effects of DGJ. This finding opens the possibility of prolonging the stabilizing effect of DGJ, reducing the frequency of administration and the inhibition of AGAL. In our opinion, the fact that potentiators can be found among drugs, such as ASA, that have been used for a long time for the chronic treatment of patients, even if their mode of action has not been elucidated, facilitates off-label usage for FD eligible patients.

## 5. Materials and Methods

### 5.1. Materials

Cell culture media and reagents were purchased from Gibco (ThermoFisher Group, Monza, Italy); fetal bovine serum (South America) and trypsin were from Euroclone (Milan, Italy); TRIzol Reagent was from ThermoFisher Scientific (Milan, Italy); QuantiTect Rev.Transcription Kit from Qiagen (Milan, Italy); SYBR Green from Biorad (Milan, Italy); cell transfection kit from InVitrogen (ThermoFisher Group, Milan, Italy).

pCMV6-AC vector encoding galactosidase alpha (*GLA*) (NM_000169) human untagged clone was purchased from Origene (Herford, Germany)and the vectors carrying individual *GLA* mutants were then obtained as described in [25].

Percoll, enzyme assay substrates and inhibitor (4-methylumbelliferyl galactopyranoside, N-acetylgalactosamine, 4-methylumbelliferyl-N-acetyl-glucosamine, cytochrome c from bovine heart, β-Nicotinamide adenine dinucleotide 2′-phosphate reduced), and lysis-M reagent for protein extraction and protease inhibitors cocktail, as well as lactosyl-sphingosine were purchased from Sigma-Aldrich (Merck, Milan, Italy); SYBR Green and Bradford reagents from Bio-Rad (Milan, Italy).

AGAL Polyclonal Antibody (PA5-27349) and GAPDH Monoclonal Antibody (MA5-15738) were purchased from ThermoFisher Scientific (Milan, Italy). Anti-mouse secondary antibody (115-035-003) was from Jackson ImmunoResearch Laboratories (Ely, UK); anti-rabbit secondary antibody (170-6515) from Bio-Rad (Milan, Italy).

Specific primers for RT-qPCR were purchased from either InVitrogen (ThermoFisher Group, Milan, Italy) (*GLA*) or Sigma-Aldrich (Merck, Milan, Italy) (*RPLP0*).

Fluorescence was detected using a Synergy HT Microplate Reader or a Varian Cary Eclipse Fluorescence Spectrometer. qRT-PCR were performed with a StepOnePlus Real-Time PCR System.

Statistical analysis and graph drawings were performed with GraphPad Prism v9. All the experiments were performed at least in biological duplicate; each biological duplicate was analyzed at least in technical duplicate. Biological replicates were considered for statistical analysis.

### 5.2. Cell Cultures and Stable Transfections

Cells were cultured in RPMI 1640 medium, supplemented with 10% fetal bovine serum, 2 mM glutamine, 0.5 mg/mL penicillin, 0.5 mg/mL streptomycin, and non-essential amino acids at 37 °C in 5% humidified CO_2_.

Patient-derived fibroblasts carrying a large deletion in *GLA* exons 3 and 4 were obtained from the Telethon Biobank and were immortalized as described by Miceli et al. [84].

Briefly, the cells were co-infected with HPV16 E6/E7 and hTERT lentiviral vectors (infection number 1). After a week, the cells were split and infected again only with hTERT (infection number 2) and cultured until stabilization.

Immortalized fibroblasts (IF) were transfected with individual pCMV6-AC plasmids carrying *GLA* mutants (IF-GLA-MUTs) or with the empty vector (IF-GLA-NULL) by electroporation. A total of 5 × 10^6^ cells from a 150 cm^2^ plate were transfected with 20 μg plasmid following the manufacturer’s instructions, then plated in a 60 cm^2^ plate with an antibiotic free medium. Forty-eight hours after the transfection, 0.1 mg/mL of geneticin was added to the medium for the selection of transfected cells. Geneticin concentration was slowly raised up to 0.4 mg/mL then brought back to 0.1 mg/mL for maintenance. Treatments with drugs were performed in the absence of geneticin.

### 5.3. Quantitative Real-Time PCR

A total of 7 × 10^5^ cells were plated in 60 cm^2^ plate and grown until 80–90% confluency. Cells were harvested in Trizol reagent and stored at −20 °C until usage. RNA was extracted according to the manufacterer’s instructions. RNA integrity was verified by electrophoresis on agarose gel, then 1 μg was reverse transcribed and 0.01 μg cDNA was analyzed. Primer sequences were 5′-TTCAAAAGCCCAATTATACAGAAA-3′ (forward) and 5′-CTGGTCCAGCAACATCAACA-3′ (reverse) for *GLA* and 5′-GACGGATTACACCTTCCCACTT-3′ (forward) 5′-GGCAGATGGATCAGCCAAGA-3′ (reverse) for *RPLP0*. The 2-ΔΔCt method [85] was used to calculate the relative mRNA expression.

### 5.4. AGAL Enzymatic Activity Assay

Cells from a 90% confluent 20 cm^2^ plate were harvested in 100 uL Roche M cOmplete lysis buffer and centrifuged at 14,000× *g* for 10 min. The enzymatic activity assay was performed as described in [25] with minor changes. A total of 40 μg of protein extract was incubated at 37 °C for 60′ in McIlvaine buffer pH 4.4 0.4 mM 4-methylumbelliferyl galactopyranoside and 8.7 mM N-acetylgalactosamine in a total volume of 55 μL using a 96 multiwell. Reaction was stopped by addition of 140 μL GlyNaOH 1 M pH 10.5 and fluorescence at 365/460 nm ex/em was read. 4-methylumbelliferone was used for the calibration curve.

### 5.5. Cell Fractionation

Cell fractionation was conducted using a self-generated Percoll gradient as described by Kominami et al. [86] and according to the manufacturer’s instructions.

Cells from confluent 450 cm^2^ were washed once in PBS, then in HB buffer (0.25 M sucrose, pH 6.8, 1 mM EDTA, 10 mM Hepes, protease inhibitors), collected with a scraper, and homogenized in HB buffer with 10 strokes of a Teflon Dounce homogenizer. The homogenate was transferred to a centrifuge tube (the final volume of the sample was 7.5 mL) and centrifuged at 1000× *g* for 5 min to remove the nuclei and the unbroken cells. A post-nuclear supernatant (PNS) was obtained. The PNS was centrifuged at 11,000× *g* for 30 min. The clear solution was removed, and the precipitate was suspended in 3.5 mL HB buffer; then, it was gently layered on 26 mL Percoll 28% (prepared in HB buffer) and centrifuged at 62,500× *g* for 100 min in a fixed-angle rotor (ultra-clear centrifuge tubes, Optima XPN-90 Ultracentrifuge, Type 70 Ti Fixed-Angle Rotor). Fractions of 2.5 mL were collected from the top of the tube; aliquots of each fraction were incubated with 0.16% Triton for 30 min, then assayed for AGAL activity and lysosomal (β-hexosaminidase) and ER (NADPH-cytochrome c reductase) markers.

An AGAL assay was performed by incubating fractions with 2.6 mM 4-methylumbelliferyl galactopyranoside and 8.7 mM N-acetylgalactosamine at 37 °C for 120 min. β-hexosaminidase activity was measured incubating fractions with 0.5 mM 4-methylumbelliferyl-N-acetyl-glucosaminide at 37 °C for 60 min. Both lysosomal activities were measured in McIlvaine buffer pH 4.4; reactions were stopped by the addition of 1 M Na_2_CO_3_; fluorescence was read at 355/460 nm ex/em. If necessary, mixtures were briefly centrifuged before reading.

NADPH-cytochrome c reductase activity was measured spectrophotometrically according to Guengerich et al. [87] and the instructions for the Cytochrome c Reductase (NADPH) Assay Kit from Sigma. Fractions were incubated with 0.42 mg/mL cytochrome c, 0.1 mM NADPH and 0.95 mM KCN in 100 mM potassium phosphate buffer pH 7.4. Absorbance at 550 nm was recorded at room temperature. The rate of change in absorbance per minute was converted to nmol/(h mL) using the extinction coefficient (ε^mM^ 21.1) for reduced cytochrome c.

### 5.6. Gb3 and Lyso-Gb3 Extraction

The extraction was accomplished according to the protocol outlined by Bligh and Dyer [88] with a few modifications. Cells from a confluent 150 cm^2^ plate were harvested with trypsin, washed with PBS, and stored as a pellet at −80 °C. Each sample was suspended in water, freeze-thawed 5 times, and the soluble proteins were measured. An appropriate amount of lactosylsphingosine was added to each sample as an internal standard (2.5 ng of standard/microg of protein).

Subsequently, chloroform, methanol, water, and hydrochloric acid up to a final condition of (1:1:1:0.05) were added in a specific sequence and with extensive mixings and breaks in between: (i) chloroform/methanol (1:2); (ii) HCl; (iii) chloroform; (iv) water. The samples were then centrifuged in glass tubes (1500 g, 45 min at 20 °C) and both the upper and the lower phases were collected (containing LysoGb3 and Gb3 respectively). The samples were eventually dried under nitrogen, then analyzed by liquid chromatography–tandem mass spectrometry.

For the UPLC-MS/MS analysis of LysoGb3 and Gb3, the chromatographic separation and MS analysis were carried out on the Q-TRAP 6500 LC-MS/MS System from AB Sciex equipped with a Shimadzu LC-20A, and the used Analyst version was 1.5.1. The mixture was separated on Luna Omega Polar from Phenomenex (1.6 μm, 100 Å, 50 × 2.1 mm). Buffer A contained 10 mM ammonium acetate in 60% water in 40% ACN and buffer B contained 10 mM ammonium acetate in 90% isopropanol and 10% ACN. The optimized UPLC protocol for separating LysoGb3, Gb3s and Lactosyl-sphingosine (used as an internal standard) is as follows: 0–2 min, 5% B; 2–7 min, 5–65% B; 7–15 min, 65–95% B; 15–18 min, 95% B; 18.1–25 min, 95–5% B; with a flow rate of 0.4 mL/min. Two independent LC-MS/MS runs were performed for each of the biological triplicate samples, using multiple reaction monitoring analyses in positive ionization mode (50-ms dwell time, current gas 40 psi, nebulizing gas of 30 psi, drying gas of 30 psi, ionspray of 5500 V, collision gas medium, and the temperature of ion source at 400 °C).

### 5.7. Proteomics Analysis

L300F total proteome was obtained as follows: control (ctrl) and ASA treated cell pellets (from 150 cm^2^ plate) were suspended in 200 μL of 8 M urea/50 mM ammonium bicarbonate (pH 8.5), 0.5% *w*/*v* sodium deoxycholate (SDC) and 1× protease inhibitor cocktail. The obtained suspensions were lysed through sonication (Vibra cell; SONICS; 1 min, 30% amplitude, 9.9 s pulses) and then centrifuged (21000 rcf, 20 min at 18 °C). The protein concentration of the supernatants was determined through a Bradford assay, and equal amounts of proteins (250 µg) were separately submitted to in-solution digestion. Briefly, proteins were reduced with 10 mM 1,4-dithiothreitol (1 h, 25 °C) and carboxy-amidomethylated with 20 mM iodoacetamide (30 min, 25 °C, in the dark). Iodoacetamide excess was then quenched with 10 mM DTT (10 min, 25 °C), urea was diluted to 1 M with 50 mM AmBic and a trypsin/Lys-C solution was added at the enzyme to proteins ratio of 1:100 *w*/*w* overnight at 37 °C. The enzymes were then quenched, formic acid (FA) was added, and the samples were dried under vacuum, dissolved in 5% FA, and equal aliquots amounts were desalted through Sep-Pak C18 1 cc (50 mg) cartridges (Waters, Milford, MA, USA). The cartridges were activated flushing 3 mL of 100% ACN and then conditioned with 3 mL of 0.1% FA (in H_2_O). The samples were then loaded, desalted flushing the cartridge with 3 mL of 0.1% FA, and finally eluted flushing two times 500 μL of 80% ACN, 20% H_2_O, 0,1% FA. The obtained peptides mixtures were dried and dissolved in 10% FA for the subsequent nano-UPLC-MS/MS analysis on a Q-Exactive Classic Mass Spectrometer coupled to an UltiMate 3000 Ultra-High-Pressure Liquid Chromatography (UPLC) system, equipped with an EASY-Spray PepMAP^TM^ RSLC C18 column (3 μm, 100 Å, 75 μm × 50 cm, ThermoFisher Scientific, Bremen). Peptides elution was achieved at a flow rate of 300 nL/min with the following gradient: 1 min at 3% B, 1 min–100 min at 45% B, 100 min–101 min at 80% B, 101 min–111 min at 80% B, 112 min back at 3% B, until 120 min (A: 0.1% AcOH, 95% H_2_O, 5% ACN; B: 0.1% AcOH, 95% ACN, 5% H_2_O). The mass spectrometer was operated in data-dependent acquisition mode. Full scan MS spectra were acquired with the following settings: scan range 375–1500 m/z, full-scan automatic gain control (AGC) target 3 × 10^6^ at 70,000 resolution, and maximum injection time 50 ms. MS2 spectra were generated for up to 10 precursors (normalized collision energy of 28%); fragment ions were acquired at a 17,500 resolution with an AGC target of 1 × 10^5^ and a maximum injection time of 50 ms. Label-free analysis was then performed scanning the raw MS files with the Proteome Discoverer software (version 2.4.1.15). MSPepSearch was used to perform a spectral library search with a mass tolerance of 10 ppm for MS1 and 0.02 Da for MS2. The target False Discovery Rate (FDR) was set to 1% (strict) and 5% (relaxed). Label-free quantification was performed exploiting both unique and razor peptides for protein abundance calculation. A pairwise ratio-based approach was used to evaluate ASA vs. control protein abundance and, for each calculated ratio, a background-based *t*-test was performed. The resulting protein abundance matrix was used for subsequent analyses.

### 5.8. Bioinformatics Analysis

Candidates for protein-ASA interactions were mined from the Biological General Repository for Interaction Datasets (BioGRID, https://thebiogrid.org/chemical/935/acetylsalicylic-acid.html; accessed date: 22 September 2021) using “acetylsalicylic acid” as the query.

We explored the Gene Expression Omnibus (GEO), looking for studies where ASA administration to human cell cultures resulted in differential gene expression. We selected the GSE58162 Series because the experimental conditions (72 h of treatment, 2.0 mM ASA) were similar to those employed in our tests. We used the interactive GEO2R tool (https://www.ncbi.nlm.nih.gov/geo/geo2r/; 1 May 2022), based on the geoquery (v2.60.0) and limma R (v 3.48.3) packages, to evaluate the effect of ASA on gene expression. 

The data in GSE58162 Series derive from three samples, “Treated with 2.0 mM aspirin” and three controls, “Untreated with aspirin”. Visual inspection of the mean-variance trends convinced us to reject the constant variance approximation and use precision weights. The trends and precision weights were calculated and visualized with the vooma() function. The logarithm of Fold Change and its significance were calculated using the eBayes() function, and the significantly differentially expressed genes (DEG) were extracted with the topTable() function. The DEG list was then intersected with two gene sets: chaperones [89] and proteasomes [90]. A slightly edited and reduced version of the R script used by GEO2R for DEG list generation and subsetting is available as part of Supplementary File S5.

### 5.9. Miscellaneous

Protein concentration was determined using the Bradford method and BSA as the standard [91].

Immunoblots were performed as in [32], using 20 μg protein extracts.

## Data Availability

Publicly available datasets were analyzed in this study. This data can be found here: [https://www.ncbi.nlm.nih.gov/geo/query/acc.cgi—accession number: GSE58162; accessed date: 1 October 2021].

## Supplementary Materials

The following supporting information can be downloaded at: https://www.mdpi.com/article/10.3390/ijms23095105/s1, File S1: comparison of the AGAL activities measured in this paper and those available in Benjamin et al. 2017; File S2: chromatogram of the Gb3 isoforms; File S3: Differential expression analysis of GSE58162 GEO dataset; File S4: List of significantly deregulated proteins from proteomics analysis; File S5: R scripts used for the analysis; File S6: Proteasome-related and molecular chaperones genes list derived from the GSE58162 GEO dataset analysis.

## Abbreviations

ABX	ambroxol
AGAL	lysosomal alpha-galactosidase
ASA	acetylsalicylic acid
DGJ	1-deoxygalactonojirimycin (Migalastat)
FD	Fabry Disease
IF-GLA-MUTs	immortalized fibroblasts transfected with individual pCMV6-AC plasmids carrying GLA mutants
IF-GLA-NULL	immortalized fibroblasts transfected with the empty vector4-PBA: 4-phenylbutyrate
