# Supplementary material for: Drug Repositioning for Fabry Disease: Acetylsalicylic Acid Potentiates the Stabilization of Lysosomal Alpha-Galactosidase by Pharmacological Chaperones
Source: Int J Mol Sci. 2022 May 4;23(9):5105. doi: 10.3390/ijms23095105 (PMC9105905; doi:10.3390/ijms23095105)
Supplement: Supplementary file 1 [file ijms-23-05105-s001.zip › File_S1.pdf]

Supplementary Table S1: Comparison of the AGAL activities measured in this paper and those available in Benjamin et al. 2017.

	This paper nmol/(h mg) ± SD	Table 1S, Benjamin et al nmol/(h mg) ± SEM	Seeman et al 2020 nmol/(h mg) ± SD
	Stable transfections in patient-derived fibroblasts not expressing *GLA*	Transient transfections in HEK-293 cells	Patient-derived fibroblasts
Empty vector	0.35±0.02	-	-
wt	5.20±0.01	-	58.5 ±31.1
D244H	0.65±0.04	6129 ± 366	-
Q280K	0.34±0.03	8019 ± 395	-
V269M	0.39±0.02	1213 ± 40	-
L300F	0.70±0.07	4746 ± 363	-
C56Y	0.47±0.02	BLD *	-
E341D	0.34±0.05	BLD *	-
A230T	0.60±0.02	BLD *	-

AGAL activities expressed as nmol/(h mg) from the independent experiments reported in this paper were averaged and compared to those reported in Benjamin et al. The results highlight that our cell lines do not overexpress AGAL. * BLD: Below the limit of detection
