# Supplementary material for: Drug Repositioning for Fabry Disease: Acetylsalicylic Acid Potentiates the Stabilization of Lysosomal Alpha-Galactosidase by Pharmacological Chaperones
Source: Int J Mol Sci. 2022 May 4;23(9):5105. doi: 10.3390/ijms23095105 (PMC9105905; doi:10.3390/ijms23095105)
Supplement: Supplementary file 1 [file ijms-23-05105-s001.zip › File_S2.pdf]

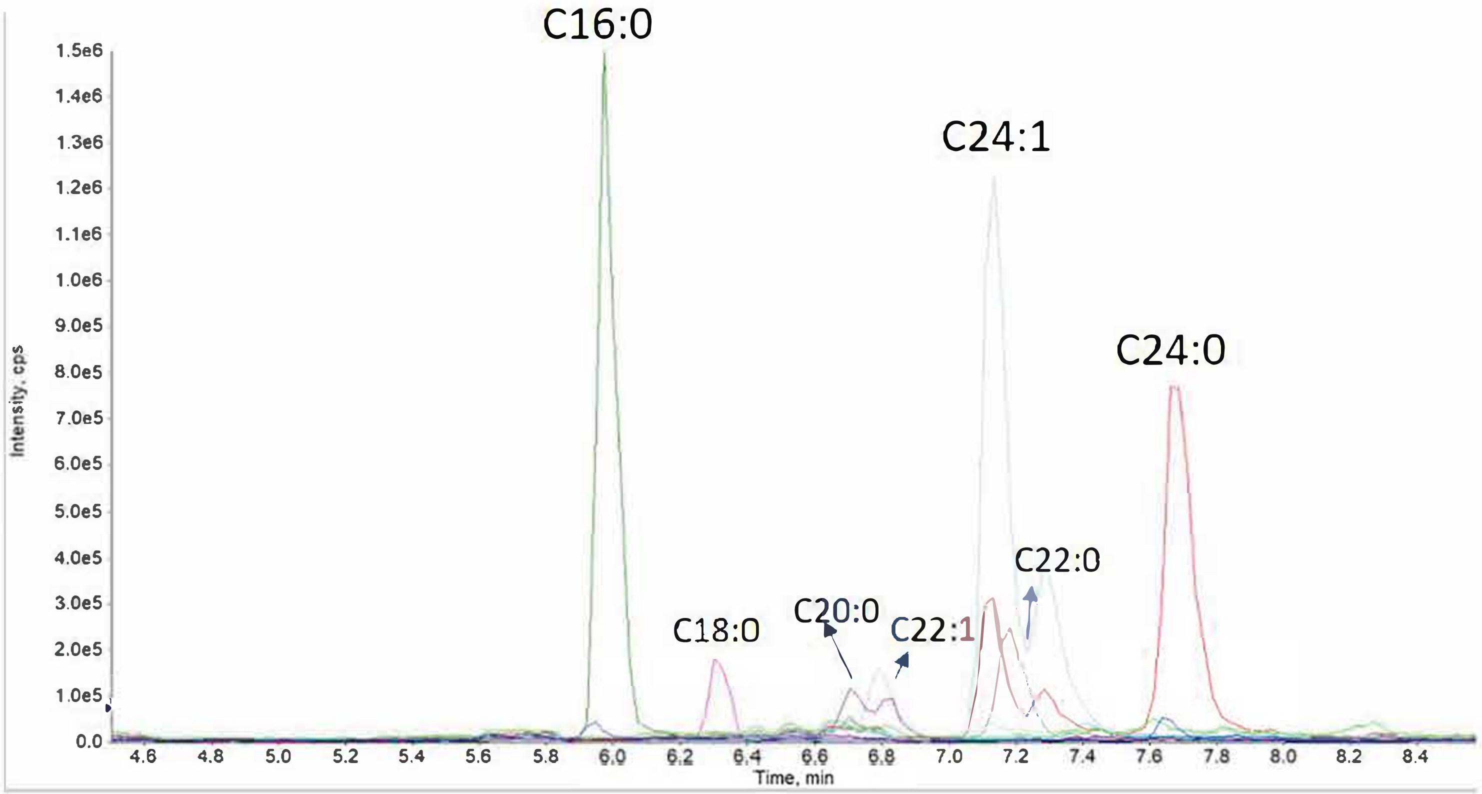

Supplementary Figure S1. Representative ion chromatograms of seven Gb3 isoforms analysed by UPLC-MS-MS.
The following transitions were monitored: 1024.4 to 844.4 m/z for C16-0-Gb3 (rt of 5.96 min); 1052.4 to 872.4 m/z for C18-0-Gb3 (rt of 6.31 min); 1080.4 to 900.4 m/z for C20-0-Gb3 (rt of 6.70 min), 1106.4 to 926.4 m/z for C22-1-Gb3 (rt of 6.71 min); 1108.4 to 928.4 m/z for C22-0-Gb3 (rt of 7.18 min); 1134.4 to 954.4 m/z for C24-1-Gb3 (rt time of 7.13 min), 1136.4 to 956.4 m/z for C24-0-Gb3 (rt of 7.67 min)
